# Marginal Fit of Chairside CAD/CAM Ceramic Inlays: An In Vitro SEM Study

**DOI:** 10.3390/dj14020098

**Published:** 2026-02-10

**Authors:** Alexandros Tzigeris, Paulína Gálfiová, Daniel Kosnáč, Andrej Thurzo, Peter Stanko

**Affiliations:** 1 Department of Stomatology and Maxillofacial Surgery, Faculty of Medicine, Comenius University in Bratislava, Heydukova 10, 812 50 Bratislava, Slovakia; thurzo3@uniba.sk (A.T.); peter.stanko@fmed.uniba.sk (P.S.); 2 Institute of Histology and Embryology, Faculty of Medicine, Comenius University in Bratislava, Sasinkova 4, 811 04 Bratislava, Slovakia; paulina.galfiova@fmed.uniba.sk (P.G.); daniel.kosnac@fmed.uniba.sk (D.K.)

**Keywords:** CAD/CAM, chairside dentistry, ceramic inlays, marginal fit, scanning electron microscopy, zirconia-reinforced lithium silicate, polymer-infiltrated ceramic network, lithium disilicate

## Abstract

**Background/Objectives:** Marginal fit is a key determinant of the clinical performance of CAD/CAM (Computer-Aided DesignComputer-Aided Manufacturing) inlay restorations. This in vitro study compared the vertical marginal gap (VMG) of three chairside CAD/CAM inlay materials—VITA Enamic, CEREC Tessera, and Celtra Duo—using scanning electron microscopy (SEM) under a standardized digital workflow. **Methods:** Standardized Class I inlay preparations were performed in 15 extracted human molars (*n* = 5/material). Restorations were fabricated using a chairside workflow (Primescan intraoral scanning, CEREC 5.3 design, Primemill milling) followed by material-specific surface treatment and cementation with a self-adhesive resin cement. VMG was measured on SEM micrographs (500× for quantitative measurements; 200× for orientation) at three sites (mesial, central, distal), with three points per site (nine points/tooth; 135 point measurements). Triplicate points were averaged to site-level means and analyzed using a linear mixed-effects model (fixed effects: material, site, material × site; random intercept: tooth), Type II ANOVA, and Tukey-adjusted pairwise comparisons. **Results:** Mean VMG values were lowest for Celtra Duo (8.09 ± 1.98 µm), followed by VITA Enamic (27.90 ± 29.76 µm) and CEREC Tessera (32.72 ± 21.80 µm). The model indicated an overall effect of material (F(2,36) = 3.51, *p* = 0.040), whereas site and material × site effects were not significant. Tukey-adjusted pairwise comparisons did not reach statistical significance. **Conclusions:** Within the standardized chairside workflow evaluated, an overall material effect on VMG was detected, but pairwise separation was inconclusive in this sample with overlapping distributions. Celtra Duo showed smaller VMG values with narrower dispersion in overall per-tooth means, while VITA Enamic and CEREC Tessera showed wider and overlapping distributions; all group means were below commonly cited clinical acceptability ranges for marginal gap.

## 1. Introduction

Digital technologies have reshaped fixed prosthodontics for indirect posterior restorations. Computer-aided design/computer-aided manufacturing (CAD/CAM) supports efficient chairside workflows and reproducible restorations while reducing reliance on conventional impressions and laboratory steps [1,2,3]. For inlays and onlays, CAD/CAM blocks are widely used because they combine aesthetics with wear resistance and chemical stability. However, current blocks vary in composition and post-processing requirements (e.g., lithium-silicate-based glass-ceramics and polymer-infiltrated ceramic networks), which may influence machining behavior and marginal integrity [4,5,6].

Marginal fit remains clinically relevant because larger marginal discrepancies can promote microleakage and biological complications. Values around ≤120 µm are commonly cited as clinically acceptable, although smaller and more consistent gaps are preferred [7,8]. In vitro studies using scanning electron microscopy (SEM) and micro-computed tomography (micro-CT) generally report acceptable marginal adaptation for lithium-silicate-based materials, but outcomes vary with both material and methodology; polymer-infiltrated/hybrid materials have shown wider dispersion in some reports [8,9,10]. In addition, evidence focused specifically on intracoronal inlay preparations is less abundant than the literature on full crowns, supporting the need for controlled comparisons in standardized inlay-type cavities [8,10]. Clinical data also suggest that digital impressions can achieve marginal fit comparable to, or better than, conventional techniques for ceramic inlays [11].

Material selection is only one part of the picture. Workflow parameters—such as scanning strategy, virtual spacer settings, milling tool condition, and post-processing procedures—can compound small inaccuracies and influence the final cavosurface margin [1,2,12,13,14,15,16]. Systematic reviews emphasize that methodological heterogeneity (including design settings, magnification, and how marginal gap is defined and measured) contributes substantially to variation between studies and can obscure material-related differences [8,10,17]. Using a unified, workflow-controlled protocol and transparently reporting key parameters (preparation geometry, cement space settings, milling and post-processing conditions, cementation procedures, and the SEM definition/sampling strategy for marginal gap) are therefore essential when comparing materials intended for single-visit chairside workflows.

Purpose and null hypothesis: Accordingly, this in vitro study compared the vertical marginal gap (VMG) of CAD/CAM inlays fabricated from Celtra Duo, CEREC Tessera, and VITA Enamic using a standardized chairside workflow and a predefined SEM sampling strategy. The null hypothesis was that VMG values would not differ (1) among materials or (2) among measurement sites (mesial, central, and distal).

## 2. Materials and Methods

### 2.1. Study Design and Ethical Considerations

This in vitro comparative study used extracted human molars (*n* = 5 teeth per material; total *n* = 15). Teeth were randomly allocated to the three material groups (*n* = 5/group) using a computer-generated random sequence. Allocation was stratified to distribute maxillary and mandibular molars evenly across groups. Both maxillary and mandibular molars were included to maximize availability of clinically relevant specimens; standardization was maintained by using the same Class I preparation design with verified dimensions for all teeth and by balancing tooth type across groups through stratified allocation. No a priori power calculation was performed; this in vitro study was designed as a workflow-controlled exploratory comparison (*n* = 5 per group), and results are interpreted primarily using effect estimates and confidence intervals rather than definitive pairwise separation. Fully anonymized teeth from a teaching collection were handled according to institutional standard operating procedures for non-interventional in vitro research and in accordance with the World Medical Association Declaration of Helsinki (2024 revision) [18]. Under institutional policy, the use of anonymized extracted teeth for in vitro studies did not require ethics committee approval or individual consent. Reporting follows the CRIS (Checklist for Reporting In Vitro Studies) guideline [19]. A sensitivity (power) analysis for a one-way ANOVA (α = 0.05, three groups) indicates that *n* = 5 teeth per group would provide approximately 80% power only for large standardized effects (Cohen’s f ≈ 0.91) [20]; detecting moderate effects would require larger groups. Accordingly, results are interpreted primarily through estimates and confidence intervals rather than definitive pairwise separation.

### 2.2. Specimen Selection and Storage

Permanent maxillary or mandibular molars with intact coronal structure (caries-free and crack-free), with no restorations and no previous endodontic treatment, were included. Both maxillary and mandibular molars were included; allocation was stratified by tooth type to balance groups. Teeth were excluded if they were third molars, premolars, or deciduous teeth, or if they exhibited caries, visible cracks/fractures, structural defects, extensive occlusal wear, existing restorations, endodontic access cavities, posts/cores, or crown defects that prevented standardized preparation. Extracted human molars were used to preserve clinically relevant enamel–dentin morphology and cavosurface characteristics, which can influence margin formation and finishing behavior [8,10]. While typodont or milled replicas can reduce biological variability, they do not replicate natural substrate structure; therefore, natural teeth were prioritized and variability was mitigated by strict inclusion criteria, standardized preparation targets, stabilization in a holding device, and random group allocation. De-identified point-level VMG measurements (Supplementary File S1), derived site-level mean datasets (Supplementary File S2), per-tooth overall mean datasets (Supplementary File S3), full statistical output (Supplementary File S4), supporting photographs/schematics (Supplementary File S5; Figures S1–S8), the Fiji/ImageJ measurement protocol and calibration notes (Supplementary File S6), and the README/data dictionary (Supplementary File S7) are provided in the Supplementary Materials.

Specimens were disinfected in 0.5% chloramine-T(CENTRALCHEM, s.r.o., Bratislava, Slovakia) for 7 days at room temperature, rinsed, soaked in distilled water for at least 2 h, and stored in 0.1% thymol (CENTRALCHEM, s.r.o., Bratislava, Slovakia) at 4 °C (solution refreshed weekly) for up to 3 months. Twenty-four hours before preparation, specimens were transferred to distilled water at 37 °C (solution refreshed once). During preparation and between procedures, specimens were kept hydrated in distilled water to minimize dehydration [21].

### 2.3. Cavity Preparation

**Figure 1:**
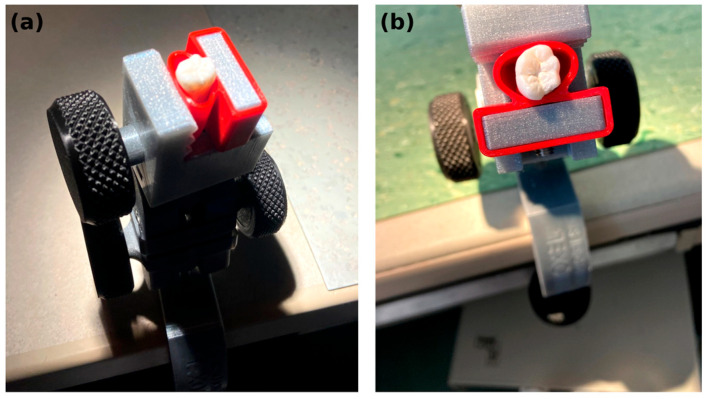
Specimen stabilization for standardized preparation orientation. (**a**) Oblique view and (**b**) occlusal view of an extracted molar secured in a holding device (TableClamp Basic, Biovoxel, Bratislava, Slovakia) and positioned with the long axis perpendicular to the base.

Standardized Class I inlay preparations were performed on the occlusal surfaces (isthmus width 2.0 ± 0.1 mm; pulpal depth 2.0 ± 0.1 mm; wall divergence 6–10°) [22]. Teeth were stabilized in a holding device (TableClamp Basic, Biovoxel, Bratislava, Slovakia) and positioned with the long axis perpendicular to the base to standardize preparation orientation. A representative photograph of the stabilization setup is shown in Figure 1.

Preparations were completed under copious water spray using flat-end straight fissure diamond burs (835/836; medium-fine, approximately 46–60 µm; Komet Dental (Gebr. Brasseler GmbH & Co. KG), Lemgo, Germany), flat-end straight carbide burs (56/57; Komet Dental (Gebr. Brasseler GmbH & Co. KG), Lemgo, Germany), and a tapered finishing diamond (847KR; approximately 25–30 µm; Komet Dental (Gebr. Brasseler GmbH & Co. KG), Lemgo, Germany). Internal line angles were rounded. Preparation depth and isthmus width were verified using a periodontal probe (Hu-Friedy Manufacturing Company, Inc., Chicago, IL, USA) and a digital caliper (Mitutoyo Corporation, Kawasaki, Kanagawa, Japan; tolerance ±0.1 mm). All preparations were performed by one experienced operator. Representative photographs of prepared specimens after completion of the Class I cavity preparation are provided in Supplementary Figure S1–S3.

### 2.4. Materials and Digital Workflow

#### 2.4.1. Materials (Microstructure and Processing Requirements)

Three chairside CAD/CAM materials were evaluated: VITA Enamic (PICN; VITA Zahnfabrik, Bad Säckingen, Germany), Celtra Duo (ZLS; Dentsply Sirona, Bensheim, Germany), and CEREC Tessera (advanced lithium disilicate; Dentsply Sirona, Bensheim, Germany) [23,24,25].

VITA ENAMIC is a polymer-infiltrated ceramic network (PICN) (“hybrid ceramic”) consisting of a dominant feldspathic ceramic network infiltrated with a polymer phase; material documentation and literature describe lower elastic modulus values than those of monolithic glass-ceramics [6,23]. Celtra Duo is a zirconia-reinforced lithium silicate (ZLS) glass-ceramic designed for chairside milling with polishing or optional glaze firing; manufacturer documentation reports higher flexural strength after the recommended firing/glazing protocol [24]. CEREC Tessera is an advanced lithium disilicate-type glass-ceramic intended for a matrix-firing step, with manufacturer documentation reporting high strength after the recommended protocol [25]. Key characteristics, post-processing steps applied in this study, and manufacturer-reported strength values are summarized in Table 1 [23,24,25].

#### 2.4.2. Digital Workflow Parameters

All restorations were fabricated within the same chairside system using a Primescan intraoral scanner (daily calibration), CEREC Software 5.3.4, a Primemill milling unit, and a SpeedFire furnace (Dentsply Sirona, Bensheim, Germany). Scanning was performed by a single operator under controlled ambient lighting using a standardized zig-zag strategy with overlapping passes. Scans were repeated if preparation margins were incompletely captured or if stitching artifacts were observed [1,2].

Design parameters were kept constant across groups. The virtual cement spacer was set to 80 µm at the margin, increasing linearly to 100 µm from 1.0 mm inside the margin. Minimum occlusal thickness was set to ≥1.5 mm and minimum marginal thickness to ≥1.0 mm. Proximal contact strength was maintained at software default settings and thickness warnings were activated. These parameters were selected to standardize cement space and design constraints across materials and align with protocols investigating the effect of cement space settings on marginal fit [13,14,15].

Restorations were milled under coolant using a roughing tool and a fine finishing tool. Milling tools were inspected before each procedure and replaced if chipped to minimize potential tool-wear bias [16]. Lithium-silicate restorations (CEREC Tessera and Celtra Duo) were fired in the SpeedFire furnace according to manufacturer schedules [24,25], whereas VITA Enamic restorations were polished. Because post-processing protocols can influence ceramic properties and marginal outcomes, processing was standardized within each material group and documented in Table 1 [12,24,25].

### 2.5. Surface Pretreatment and Cementation

Restoration pretreatment followed material-specific protocols. For VITA ENAMIC (polymer-infiltrated ceramic network), the intaglio surface was etched with 5% hydrofluoric acid (IPS Ceramic Etching Gel, Ivoclar Vivadent AG, Schaan, Liechtenstein) for 60 s, rinsed thoroughly with water, air-dried, and treated with silane (Monobond Plus, Ivoclar Vivadent AG, Schaan, Liechtenstein) for 60 s [26]. For Celtra Duo and CEREC Tessera (lithium-silicate ceramics), the intaglio surface was etched with 5% hydrofluoric acid for 30 s, rinsed thoroughly with water, air-dried, and treated with silane for 60 s [12].

Tooth pretreatment consisted of selective enamel etching. Enamel margins were etched with 37% phosphoric acid (Total Etch, Ivoclar Vivadent AG, Schaan, Liechtenstein) for 15 s, rinsed for 30 s, and gently air-dried; dentin was not etched and was maintained slightly moist prior to cementation.

**Figure 2:**
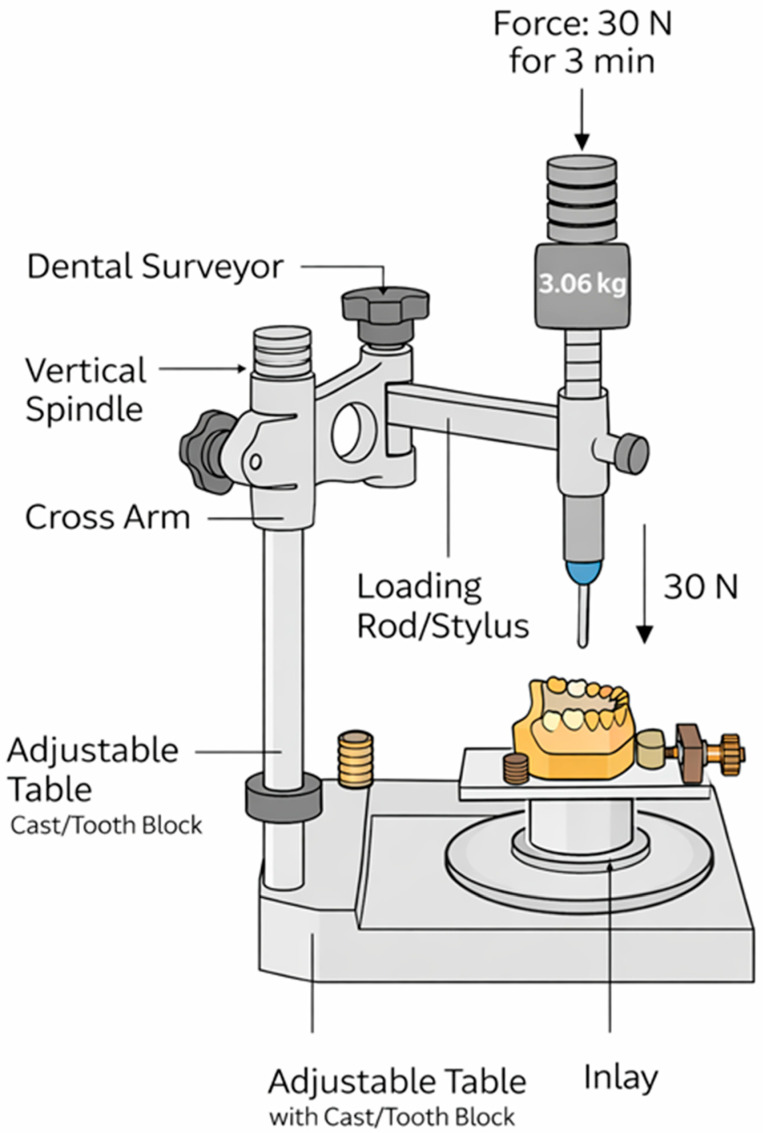
Standardized cementation setup used to apply a constant axial seating force. A dental surveyor (Saeshin Precision XO Ltd.) was used to align a vertical loading rod/stylus over the occlusal surface of the inlay. A calibrated 3.06 kg mass generated an axial load of approximately 30 N, which was maintained for 3 min during cementation.

Inlays were luted with Panavia SA Cement Universal (self-adhesive resin cement; Kuraray Noritake Dental Inc., Okayama, Japan) using a standardized seating protocol. Immediately after placement, a constant axial seating force of 30 N was applied for 3 min using a dental surveyor (Saeshin Precision XO Ltd., Daegu, Republic of Korea) configured as a loading device (Figure 2). The tooth block was stabilized on the surveyor’s adjustable table and oriented so that the long axis of the prepared tooth was vertical. A rigid, large-diameter cylindrical loading rod/stylus from the surveyor accessory set was positioned centrally on the occlusal surface of the inlay to deliver a purely axial load and minimize lateral components. The seating force was generated by a calibrated mass of 3.06 kg (approximately 30 N; F = m·g, g = 9.81 m/s^2^) applied to the loading assembly and maintained continuously for the specified duration. All cementations were performed by a single operator using identical procedures across groups.

Excess cement was removed immediately after seating using microbrushes and an explorer along the cavosurface margin, followed by flossing of proximal embrasures where accessible. Margins were re-inspected under magnification to confirm that visible excess cement had been eliminated before final curing. Margins were then coated with glycerin gel (Liquid Strip, Ivoclar Vivadent AG, Schaan, Liechtenstein) to minimize oxygen inhibition. Light-curing was performed for 20 s per surface with the curing tip positioned as close as possible to the restoration surface and oriented perpendicular to each surface. A high-intensity multiwavelength LED light-curing unit (VALO Cordless, Ultradent Products Inc., South Jordan, UT, USA; approximately 1200 mW/cm^2^; emission 430–480 nm) was used for all polymerization procedures [26,27,28]. Batch/lot numbers for all materials were recorded. Cement composition parameters (e.g., filler characteristics) were not analyzed in this study. Representative photographs of specimens after inlay luting, cleanup, glycerin-gel application, and completion of light-curing are provided in Supplementary Figures S6–S8.

### 2.6. Storage Prior to Evaluation

After cementation and cleanup, specimens were stored fully immersed in distilled water at 37 °C for 24 h before SEM imaging and marginal gap assessment. This storage standardized post-cementation conditions and allowed the resin cement to reach a stable set under moist, physiologic-temperature conditions before SEM assessment. No thermocycling or mechanical aging was performed prior to marginal fit assessment. Specimen storage conditions were selected based on prior recommendations for extracted teeth [21].

### 2.7. SEM Imaging and Measurement Protocol

**Figure 3:**
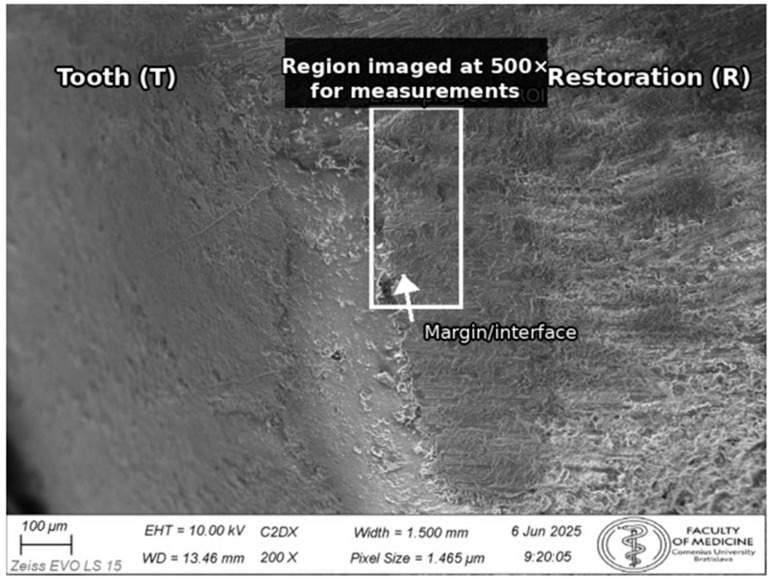
Representative SEM overview image (200×) used for orientation and site localization prior to quantitative assessment. Quantitative marginal gap measurements were performed on 500× micrographs in ImageJ (1.54p).

Specimens were sputter-coated with Au/Pd (Quorum Technologies Ltd., Laughton, East Sussex, UK) (approximately 10 nm; approximately 30 mA for 60 s) and imaged using a Zeiss EVO LS 15 scanning electron microscope (Zeiss, Oberkochen, Germany) in high-vacuum secondary electron mode (accelerating voltage 10 kV; stage tilt 0°). Working distance was adjusted to achieve optimal focus (approximately 9–11 mm). For documentation and orientation, images were acquired at 200×; all quantitative measurements were performed on 500× micrographs. Representative photographs of specimens after Au/Pd coating are shown in Supplementary Figures S4 and S5. A representative 200× overview image used for orientation and site localization is shown in Figure 3.

**Figure 4:**
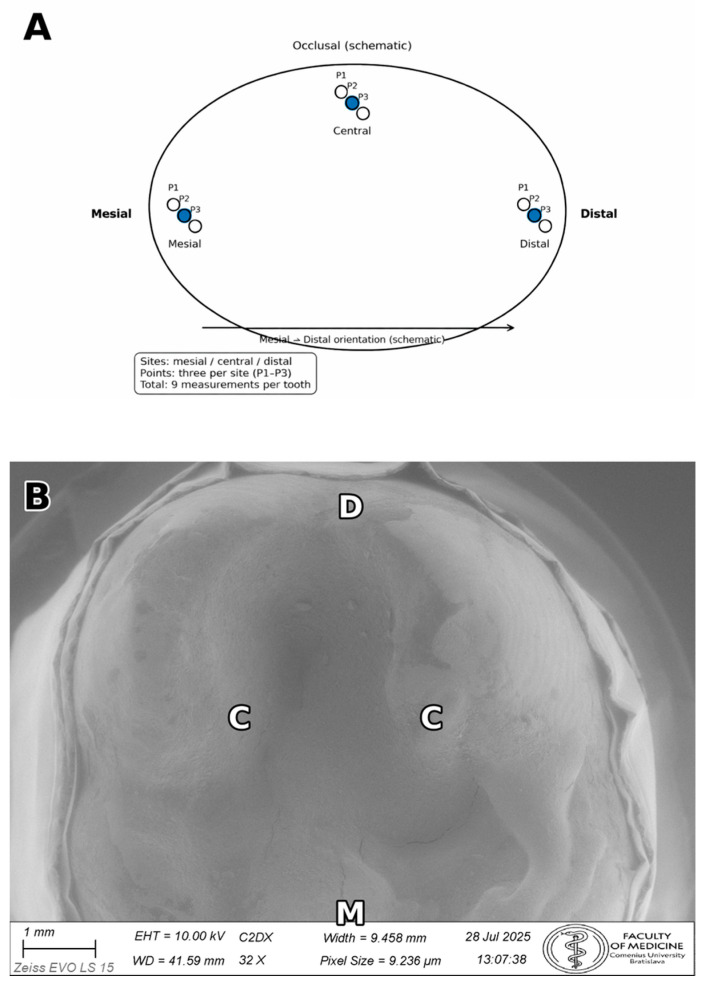
Definition of measurement sites and points along the cavosurface margin. (**A**) Schematic definition of three sites (mesial, central, distal) and three points per site (P1–P3), yielding nine point measurements per tooth used to compute site-level means. (**B**) Low-magnification SEM overview illustrating the predefined measurement sites along the cavosurface margin: mesial (M), central (C), and distal (D) (representative specimen). These sites were subsequently imaged at higher magnification (500×) for quantitative VMG measurements.

Measurements were performed in Fiji (2.17.0)/ImageJ (1.54p) after calibration to each image’s scale bar. The detailed Fiji/ImageJ calibration and measurement workflow is provided in Supplementary File S6. The primary metric was the vertical marginal gap (VMG), defined as the perpendicular distance from the restoration margin to the tangent of the tooth finish line (cavosurface margin) at the measurement point. Absolute marginal discrepancy (AMD)—which incorporates both vertical discrepancy and horizontal over-/under-extension—was not evaluated because reliable identification of the horizontal component is not consistently possible on external SEM images without sectioning; therefore, a single, reproducible VMG definition was used for all specimens [17]. After cleanup, luting cement at the external margin may be flush with the interface and not readily distinguishable in secondary electron images; measurements were therefore made at the tooth and restoration margins as defined above. For each tooth, three predefined sites were evaluated (mesial, central, distal). The definition of sites and measurement points is illustrated in Figure 4.

Sites were defined as the mesial and distal cavosurface margins and the central occlusal margin at the midpoint of the preparation. At each site, three measurement points were recorded (nine points/tooth; total 135 point measurements). Measurements were made using the straight-line tool, with the measurement line drawn perpendicular to the local cavosurface tangent at the selected point. If either margin could not be identified unambiguously at 500×, the area was re-imaged and the measurement repeated on a newly acquired micrograph.

Calibration was verified at the start of each measurement session by measuring the SEM scale bar (tolerance ±1 µm at 500×). To evaluate repeatability, 15% of measurement points were re-measured at least 7 days later by the same examiner while blinded to material (micrographs were coded and contained no material identifiers), yielding an ICC (3,1) of 0.97 (95% CI: 0.94–0.99) [29]. All de-identified point-level measurements and derived datasets are provided in Supplementary Files S1–S3, and the full statistical output is provided in Supplementary File S4. SEM images were exported without enhancement; figure labels and illustrative measurement lines were added using image annotation software without altering the underlying image content.

### 2.8. Statistical Analysis

Vertical marginal gap (VMG, µm) was the dependent variable. For each tooth, the three points measured at each site were averaged to obtain site-level means (mesial, central, distal). A linear mixed-effects model was then fitted to evaluate the effects of material, site, and their interaction as fixed effects, with a random intercept for tooth to account for repeated site measurements within the same specimen. All analyses were performed on coded datasets to maintain blinding until completion of measurements. Overall tests of fixed effects were performed using Type II ANOVA. Model-based estimated marginal means (EMMs; adjusted means) with 95% confidence intervals were derived to summarize differences between materials averaged across sites, and Tukey-adjusted pairwise comparisons were used for between-material contrasts (α = 0.05). Model diagnostics (Q–Q plot and residuals versus fitted values) did not indicate major departures from model assumptions. Analyses were performed in Python (v3.11.2) using Statsmodels (v0.14.3) and SciPy (v1.14.1). Given the small sample size (*n* = 5 teeth/group), interpretation emphasizes effect direction and uncertainty (confidence intervals) rather than definitive pairwise separation [10].

## 3. Results

### 3.1. Descriptive Outcomes

**Figure 5:**
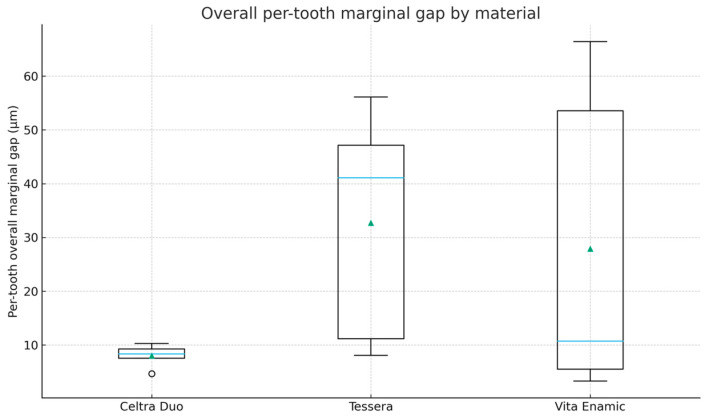
Overall per-tooth VMG (µm) by material (*n* = 5 teeth/group). Boxplot shows median and interquartile range; mean is indicated.

**Figure 6:**
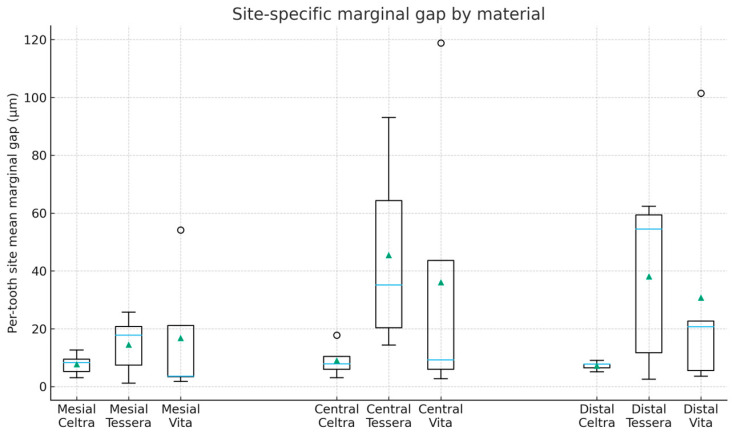
Per-tooth site means of VMG (µm) by material and site (*n* = 5 teeth/group). Boxplots are shown for mesial, central, and distal sites.

Per-tooth site-level mean vertical marginal gap (VMG, µm) values are summarized in Table 2 (*n* = 5 teeth per material). Overall per-tooth mean VMG values by material (averaged across mesial, central, and distal sites) are presented in Table 3 and visualized in Figure 5, while site-specific distributions are shown in Figure 6. The overall mean VMG was lowest for Celtra Duo (8.09 ± 1.98 µm), followed by VITA Enamic (27.90 ± 29.76 µm) and CEREC Tessera (32.72 ± 21.80 µm). Differences in apparent dispersion between Figure 5 and Figure 6 are expected because Figure 5 summarizes per-tooth means averaged across all three sites, whereas Figure 6 displays site-level per-tooth means; site-specific variability (particularly at the central site) can therefore appear more pronounced.

VITA Enamic and CEREC Tessera showed larger standard deviations, indicating greater between-specimen variability. Given the small group size (*n* = 5 teeth per material), SDs are sensitive to between-tooth differences and occasional higher site means; therefore, distributions (Figure 5 and Figure 6) and model-based estimates with confidence intervals are emphasized alongside mean ± SD. For transparency, de-identified point-level VMG measurements are provided in Supplementary File S1, with derived site-level and overall mean datasets in Supplementary Files S2 and S3, and the statistical output in Supplementary File S4.

### 3.2. Inferential Outcomes

A linear mixed-effects model (VMG ~ material × site + (1|tooth)) was fitted to evaluate the effects of material, site, and their interaction while accounting for repeated site measurements within each tooth. Type II tests indicated a statistically significant overall effect of material on VMG, while the effects of site and the material × site interaction were not significant: material F(2,36) = 3.51, *p* = 0.040; site F(2,36) = 1.60, *p* = 0.215; material × site F(4,36) = 0.43, *p* = 0.786.

Model-based estimated marginal means (EMMs; averaged over sites) were 8.09 µm (95% CI 6.07–10.12) for Celtra Duo, 27.90 µm (95% CI 7.41–48.39) for VITA Enamic, and 32.72 µm (95% CI 17.47–47.97) for CEREC Tessera. Tukey-adjusted pairwise comparisons between materials did not reach statistical significance (all *p* ≥ 0.20). Accordingly, the inferential results support an overall material-related difference in VMG, but do not provide conclusive evidence for any specific between-material pairwise separation under multiplicity adjustment in this exploratory sample.

**Figure 7:**
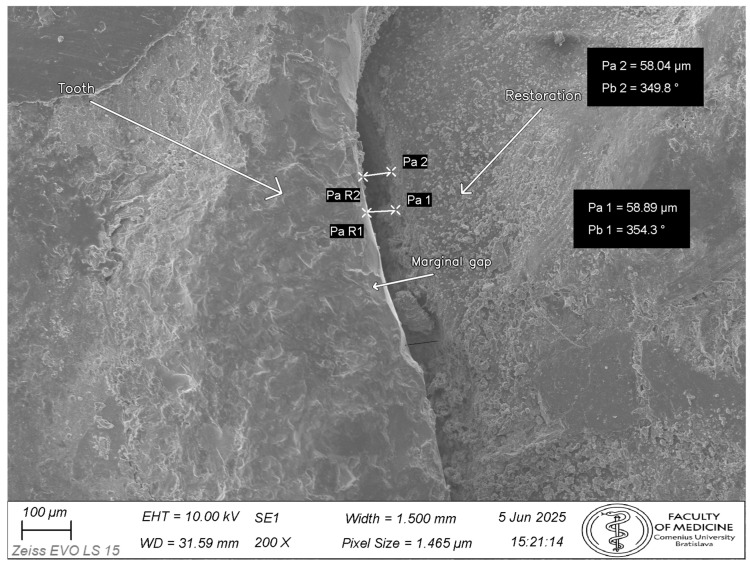
Representative SEM micrograph (200×) illustrating orientation and the vertical marginal gap (VMG) measurement principle at the tooth–restoration margin. Tooth structure (T) and restoration (R) are indicated, and the VMG is shown by arrows as the perpendicular distance from the restoration margin to the local tangent of the tooth finish line at the measurement point. In the on-image measurement labels, Pa denotes the measured VMG distance (µm) at the indicated site (Pa1, Pa2), Pb denotes the corresponding angle (°) of the local tangent/reference used to define the perpendicular (Pb1, Pb2), and PaR1/PaR2 indicate the corresponding restoration-margin points used for each measurement. This image is provided for orientation and demonstration of the measurement geometry; all quantitative measurements were performed on 500× SEM images at predefined sites/points. Scale bar: 100 μm.

**Figure 8:**
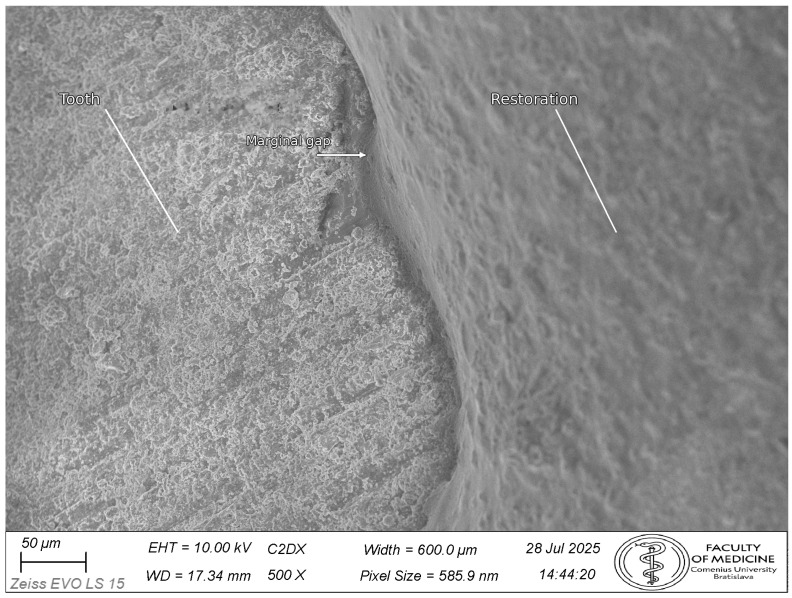
Representative SEM micrograph (500×) of the tooth–restoration margin (CEREC Tessera group). Tooth structure (T), restoration (R), and the marginal gap (VMG) are indicated. VMG was quantified in Fiji/ImageJ as the perpendicular distance to the local tooth tangent at the finish line, following the standardized protocol and point distribution described in Figure 4. Scale bar: 50 μm.

**Figure 9:**
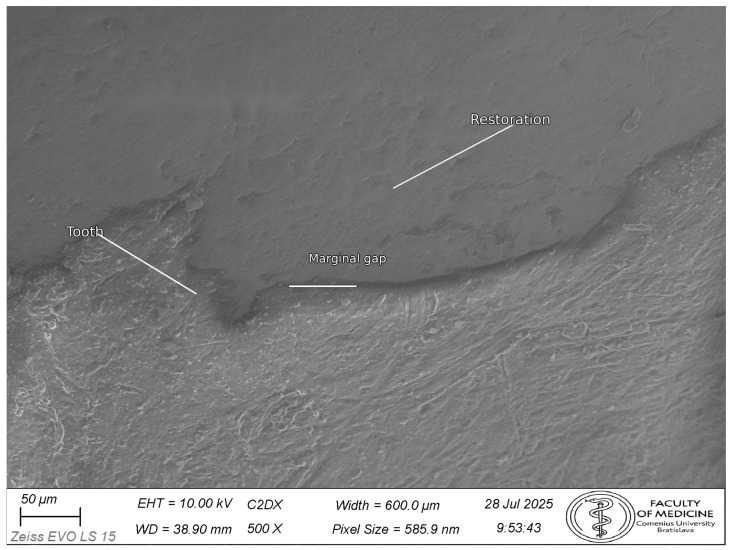
Representative SEM micrograph (500×) of the tooth–restoration margin (VITA Enamic group). Tooth structure (T), restoration (R), and the marginal gap (VMG) are indicated. VMG was measured in Fiji/ImageJ as the perpendicular distance from the restoration margin to the tangent of the tooth finish line at the measurement point. Scale bar: 50 μm.

Representative SEM micrographs are shown in Figure 7, Figure 8 and Figure 9 (Figure 7: 200× orientation image, scale bar 100 µm; Figure 8 and Figure 9: 500×, scale bar 50 µm).

## 4. Discussion

This in vitro study compared the vertical marginal gap (VMG) of chairside CAD/CAM inlays fabricated from three CAD/CAM block materials using a standardized digital workflow. An overall effect of material on VMG was detected in the mixed-effects model, while site and the material × site interaction were not significant. Although Tukey-adjusted pairwise comparisons did not reach statistical significance, the descriptive pattern showed smaller VMG values for Celtra Duo and wider, overlapping distributions for VITA Enamic and CEREC Tessera. Because group sizes were small, the findings are presented primarily as estimates of magnitude and dispersion under a standardized workflow.

Several steps were taken to limit methodological heterogeneity and isolate material-related differences. All restorations were produced within one chairside ecosystem using the same scanner (daily calibration), software version, spacer settings, milling unit, and standardized post-processing following each manufacturer’s protocol. Preparations were performed with fixed geometric targets and verified with a caliper, and specimens were stabilized in a standardized holding device to maintain consistent orientation. Cementation variables were standardized (surface pretreatment protocols, seating force, cleanup timing, and curing approach). SEM imaging used fixed acquisition conditions and a predefined VMG measurement definition at standardized sites and points. Milling tools were inspected before each procedure and replaced if chipped to minimize potential tool-wear bias [16]. Data were analyzed using a mixed-effects model to account for clustering of repeated measurements within each tooth.

Previous in vitro inlay/onlay studies report a wide range of marginal fit outcomes, and systematic reviews emphasize that results depend strongly on preparation design, spacer settings, cementation protocol, and the definition and method used to quantify marginal discrepancy [8,17]. Micro-CT studies evaluating ceramic inlays also report that fit differs between manufacturing approaches and CAM systems, supporting the need for workflow-controlled comparisons [22]. In clinical evaluation, ceramic inlays produced from digital impressions have shown marginal fit comparable to, or better than, conventional approaches [11]. Consistent with these observations, the present study aimed to reduce between-study variability by applying a unified scan–design–mill–post-process–lute workflow within a single chairside system, so that observed differences are more likely to reflect a combination of material-related behavior and material-specific post-processing steps rather than uncontrolled workflow parameters.

The tendency toward smaller VMG values for Celtra Duo is plausible given differences in material structure and post-processing. Lithium-silicate ceramics undergo crystallization/glazing procedures that may influence margin quality and dimensional stability, and marginal outcomes may be sensitive to furnace parameters and finishing steps [4,12]. In contrast, polymer-infiltrated ceramic network (PICN) materials are finished by polishing rather than crystallization and may be more technique-sensitive in finishing and margin refinement, which can contribute to wider dispersion in VMG outcomes [9]. Consistent with this, three-dimensional studies have shown that manufacturing and post-processing procedures can influence both marginal and internal adaptation [30]. Importantly, these factors act alongside workflow parameters—scanner strategy and calibration, spacer settings, milling bur condition, and thermal schedules—which are recognized determinants of fit and should be standardized and reported for meaningful comparisons [1,13,15].

Across sites, central values tended to be higher than mesial and distal values; however, the site effect was not statistically significant. This pattern may relate to occlusal morphology and scan incidence angles that influence margin capture and model stitching, as well as seating dynamics during cementation under load [1,19,21]. Because site-related differences were not statistically supported, these observations should be interpreted cautiously.

Clinical implications. Within the standardized chairside workflow used (including an 80–100 µm spacer design, controlled milling, and material-specific post-processing), all three materials achieved mean VMG values within commonly cited clinical acceptability ranges [7,8]. While the results descriptively favored Celtra Duo in terms of smaller VMG values and narrower dispersion, the overlap between groups indicates that workflow control and consistent processing may be as important as material choice for achieving predictable margins in routine practice [1,13,15]. This is consistent with clinical reports comparing digital and conventional workflows for ceramic inlays [11]

Strengths and limitations. Strengths include a unified chairside workflow within a single system, standardized Class I preparation parameters, controlled seating force during cementation, and SEM-based VMG measurements with high repeatability (ICC). Limitations include the in vitro setting, the small sample size (*n* = 5/group), and the absence of thermomechanical aging, which restricts extrapolation to long-term clinical performance. The small sample size without an a priori power calculation limits statistical power and contributes to wide uncertainty around between-material differences; accordingly, the findings should be interpreted as exploratory and hypothesis-generating. As a sensitivity analysis indicates, *n* = 5 teeth per group provides adequate power only for large between-material effects; smaller, potentially clinically relevant differences may therefore have gone undetected [20]. Because *n* = 5 per group, dispersion estimates (SD) are inherently unstable and may be influenced by specimen-level variability typical of extracted teeth and intracoronal preparations. Although printed or milled replicas could further reduce operator-dependent variability, natural teeth were chosen for clinical substrate realism; preparation variability remains a potential contributor to dispersion despite standardized targets and verification. Including both maxillary and mandibular molars may have introduced additional anatomical variability; although tooth type was balanced across groups, future studies could restrict specimens to a single molar type to further reduce heterogeneity. The study was also limited to Class I preparations and one CAD/CAM ecosystem, so outcomes may differ with other preparation designs, scanners, milling units, or firing/polishing protocols. Finally, VMG assessment was based on external SEM measurements at predefined sites and does not provide three-dimensional information on internal adaptation; complementary 3D methods could be considered in future work [30].

Overall, under the standardized chairside workflow evaluated, all three materials achieved low mean VMG values. The results suggest smaller VMG values for Celtra Duo, with wider and overlapping distributions for VITA Enamic and CEREC Tessera, although pairwise separation was inconclusive in this sample.

Future studies should confirm these workflow-controlled findings in larger samples and under clinically relevant aging protocols (thermocycling and mechanical fatigue), and should evaluate additional preparation designs and CAD/CAM ecosystems. From a practical standpoint, the present results reinforce that workflow standardization (preparation geometry, digital parameters such as cement space, seating force, cleanup, and measurement definitions) is critical for reproducible marginal adaptation assessments in chairside inlays, alongside any material-related differences. More broadly, recent reviews in complex oral rehabilitation emphasize the importance of standardized protocols and advanced digital planning approaches to improve reproducibility and clinical outcomes across dental workflows [31].

## 5. Conclusions

Under the standardized chairside CAD/CAM workflow evaluated, the overall analysis indicated that vertical marginal gap differed among materials, with Celtra Duo showing the smallest mean values. Pairwise differences were not significant in this small, workflow-controlled exploratory sample, and variability was greater in the VITA Enamic and CEREC Tessera groups. All mean gap values remained within commonly cited clinical acceptability ranges.

## Figures and Tables

**Table 1:** Key characteristics and manufacturer-reported flexural strength of the tested chairside CAD/CAM blocks and post-processing steps used in this study. Flexural strength values are reported from manufacturer technical documentation under stated conditions (e.g., polished vs. fired) [23,24,25].

Material	Material Class/Microstructure	Post-Processing in This Study	Reported Flexural Strength (MPa)	Source/Notes
VITA ENAMIC (PICN)	Polymer-infiltrated ceramic network (PICN; “hybrid ceramic”: ceramic network infiltrated with polymer)	Polished	150–160 ^1^	Manufacturer technical data; polymer component and lower elastic modulus may increase sensitivity to bonding protocol and finishing [23]
Celtra Duo (ZLS)	Zirconia-reinforced lithium silicate glass-ceramic (ZLS)	Glaze-fired per manufacturer schedule	370 ^2^	Manufacturer technical data; optional glazing/firing is associated with higher reported strength and may influence interpretation of marginal adaptation [24].
CEREC Tessera (ALD)	Advanced lithium disilicate-type glass-ceramic (lithium disilicate + additional crystalline phase)	Matrix-fired per manufacturer schedule	>700 ^3^	Manufacturer technical data; high reported post-firing strength, but marginal outcomes can still depend on preparation design and protocol adherence [25].

Conditions: ^1^ Polished (no firing). ^2^ After glaze firing; manufacturer also reports 210 MPa when polished (no firing). ^3^ After recommended matrix firing; reported as biaxial flexural strength in manufacturer documentation.

**Table 2:** Per-tooth site means (µm) by material (mean ± SD; *n* = 5 teeth per material).

Material	Mesial (Mean ± SD)	Central (Mean ± SD)	Distal (Mean ± SD)
Celtra Duo	7.75 ± 3.73	9.29 ± 5.28	7.24 ± 1.50
CEREC Tessera	14.57 ± 10.04	45.48 ± 32.91	38.12 ± 28.59
VITA Enamic	16.82 ± 22.31	36.09 ± 49.12	30.78 ± 40.42

**Table 3:** Per-tooth overall means (µm) by material (mean ± SD; *n* = 5).

Material	Overall Mean ± SD
Celtra Duo	8.09 ± 1.98
CEREC Tessera	32.72 ± 21.80
VITA Enamic	27.90 ± 29.76

## Data Availability

The original contributions presented in this study are included in the article and Supplementary Materials. Further inquiries can be directed to the corresponding author. The de-identified raw point-level marginal gap measurements (CSV/XLSX), derived site-level and per-tooth mean datasets (CSV/XLSX), and a blinded README/data dictionary are provided in the Supplementary Materials. The analysis code (Python/statsmodels) and figure-generation scripts, together with a statistical output report and the ImageJ measurement protocol, are also included in the Supplementary Materials.

## Supplementary Materials

The following supporting information can be downloaded at: https://www.mdpi.com/article/10.3390/dj14020098/s1. File S1_Data: De-identified point-level marginal gap measurements (CSV). File S2_SiteMeans: Derived site-level mean datasets (mesial, central, distal) (CSV). File S3_OverallMeans: Derived per-tooth overall mean datasets (CSV). File S4_StatsReport: Statistical output report (TXT). File S5_SupplementaryFigures: Supplementary Figures S1–S3 (Figure S1: schematic definition of measurement sites and point placement; Figure S2a–c: specimen mounting in the custom holder; Figures S1–S8 (TIF): SEM setup and specimen positioning). File S6_Methods_ImageJ: ImageJ/Fiji measurement protocol and calibration notes (TXT). File S7_README_DataDictionary: README file and blinded data dictionary (TXT).

## Abbreviations

ALD	advanced lithium disilicate
ANOVA	analysis of variance
CAD/CAM	computer-aided design/computer-aided manufacturing
CI	confidence interval
EMM	estimated marginal mean
VMG	vertical marginal gap
PICN	polymer-infiltrated ceramic network
SD	standard deviation
SEM	scanning electron microscopy
ZLS	zirconia-reinforced lithium silicate
