# Supplementary figures and images for: Marginal Fit of Chairside CAD/CAM Ceramic Inlays: An In Vitro SEM Study
Source: Dent J (Basel). 2026 Feb 10;14(2):98. doi: 10.3390/dj14020098 (PMC12938975; doi:10.3390/dj14020098)

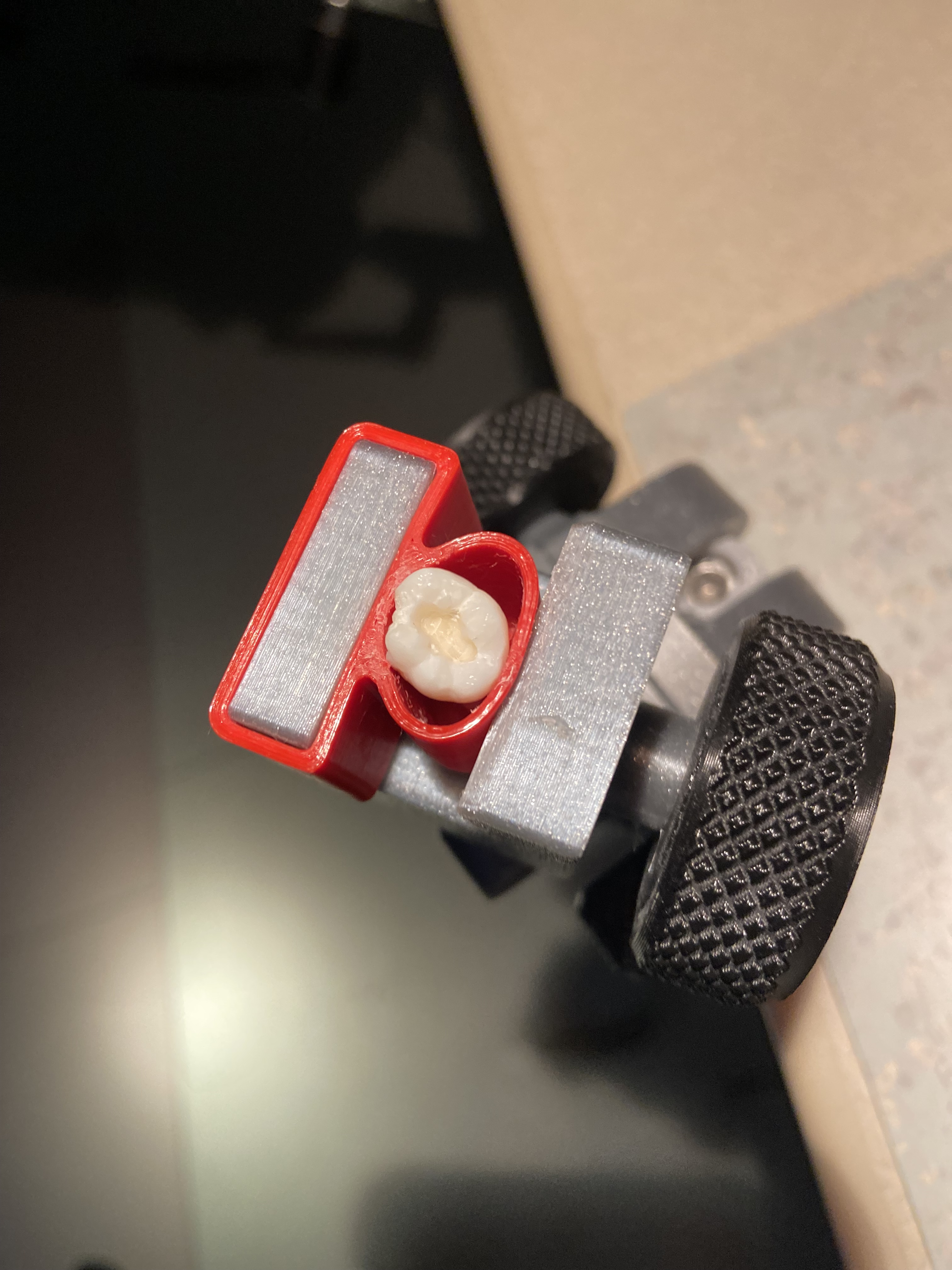

Supplement: Supplementary file 1 [file dentistry-14-00098-s001.zip › S5_SupplementaryFigures/FigureS1.tif]

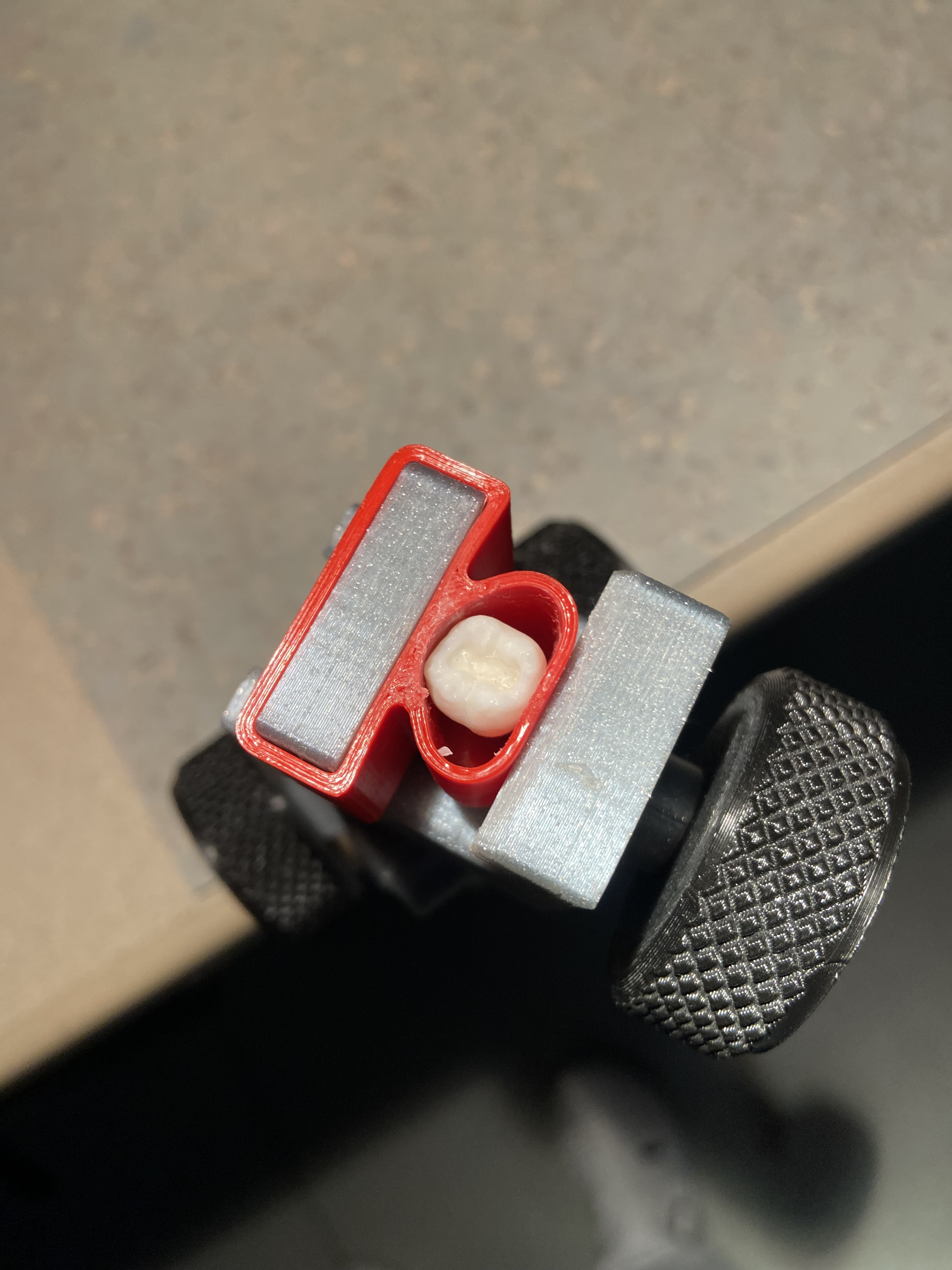

Supplement: Supplementary file 1 [file dentistry-14-00098-s001.zip › S5_SupplementaryFigures/FigureS2.tif]

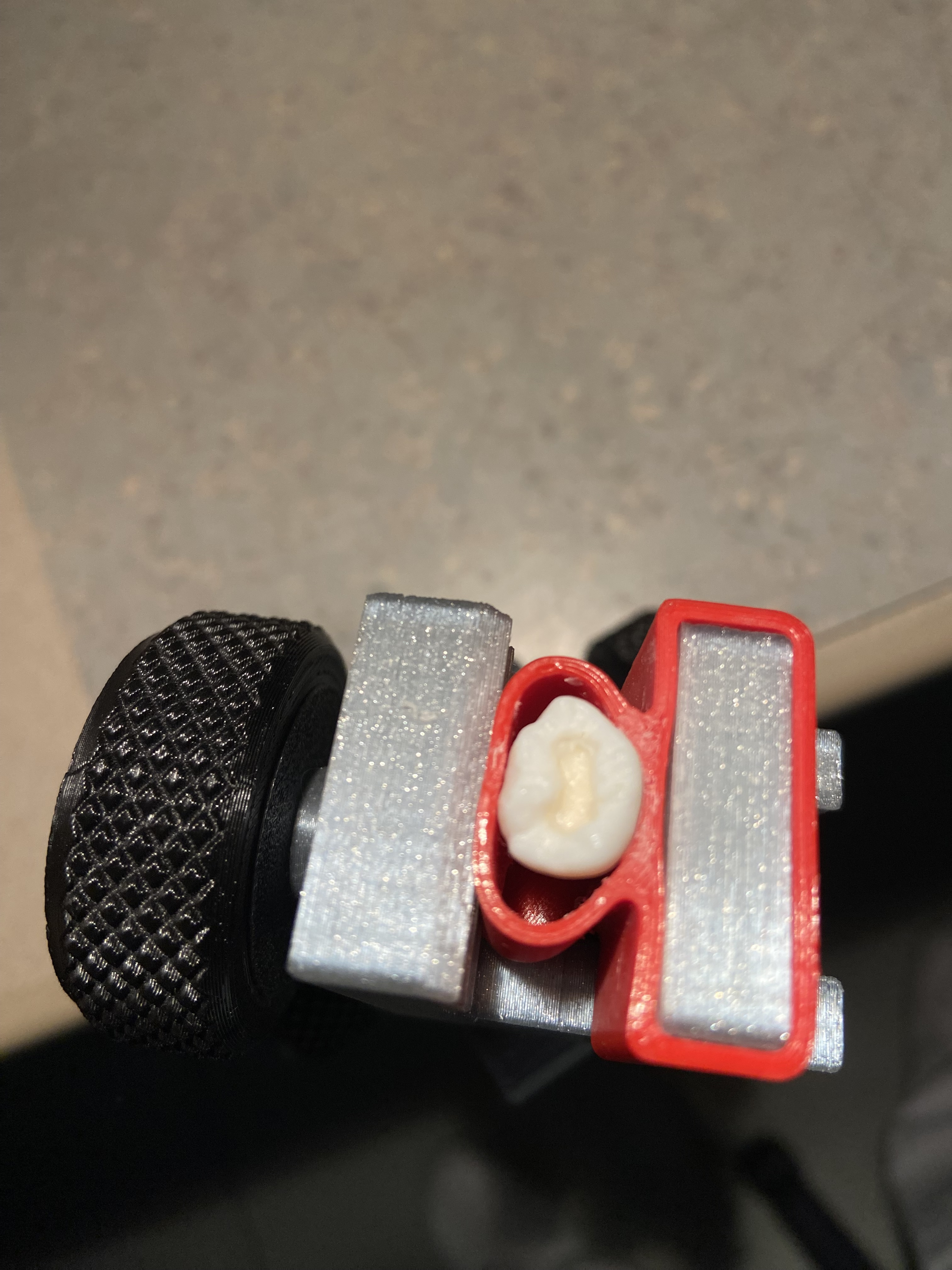

Supplement: Supplementary file 1 [file dentistry-14-00098-s001.zip › S5_SupplementaryFigures/FigureS3.tif]

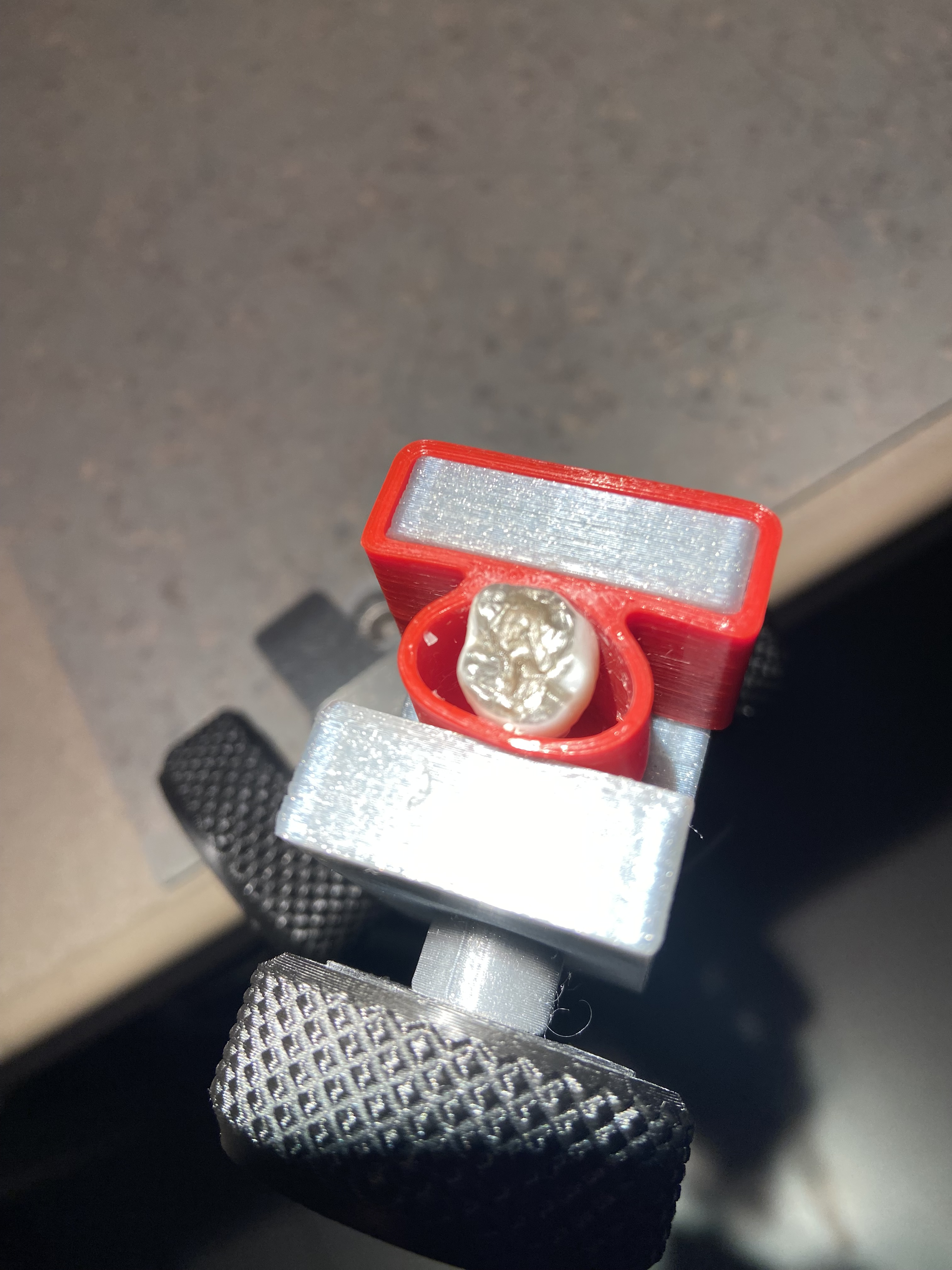

Supplement: Supplementary file 1 [file dentistry-14-00098-s001.zip › S5_SupplementaryFigures/FigureS4.tif]

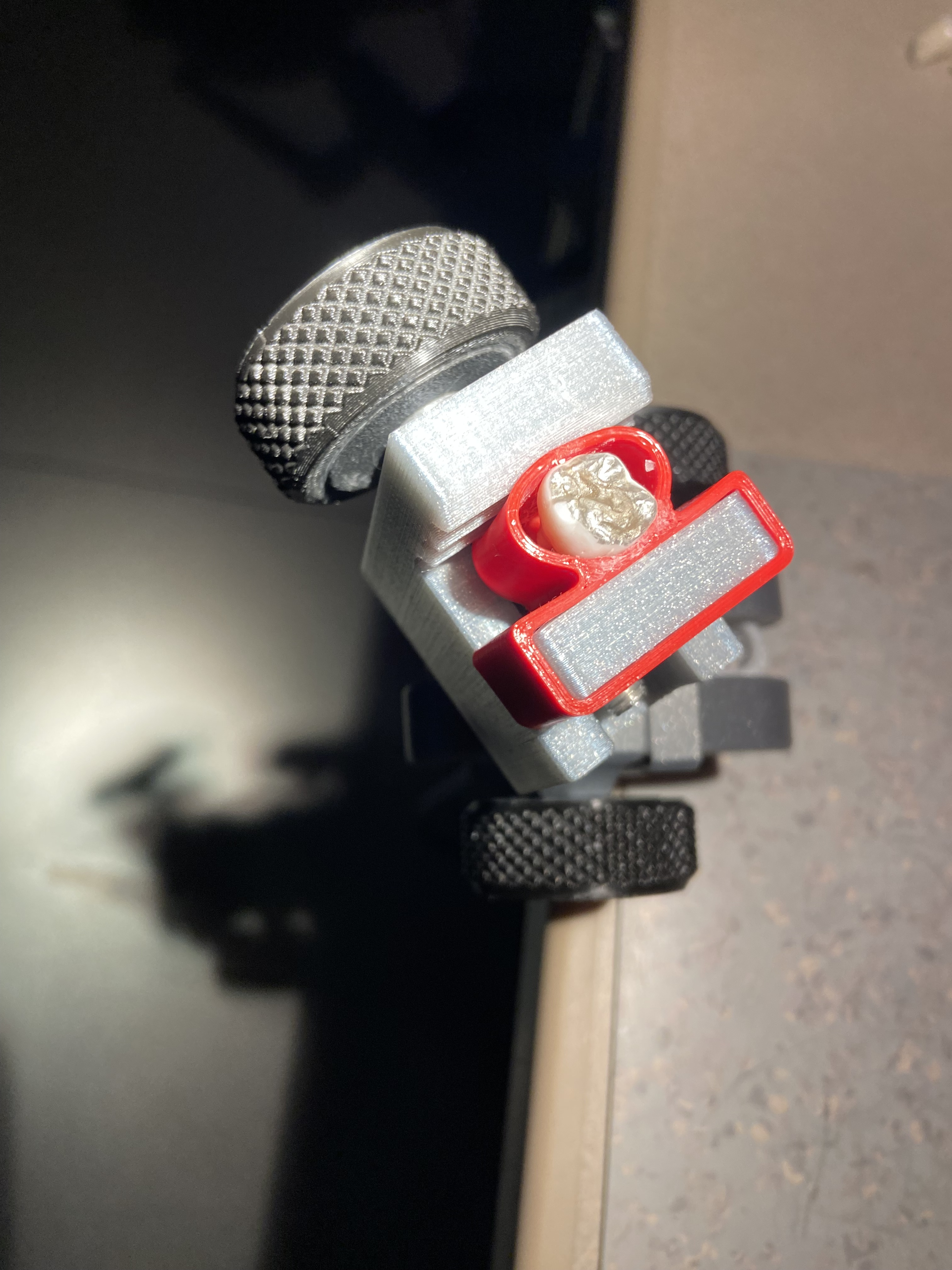

Supplement: Supplementary file 1 [file dentistry-14-00098-s001.zip › S5_SupplementaryFigures/FigureS5.tif]

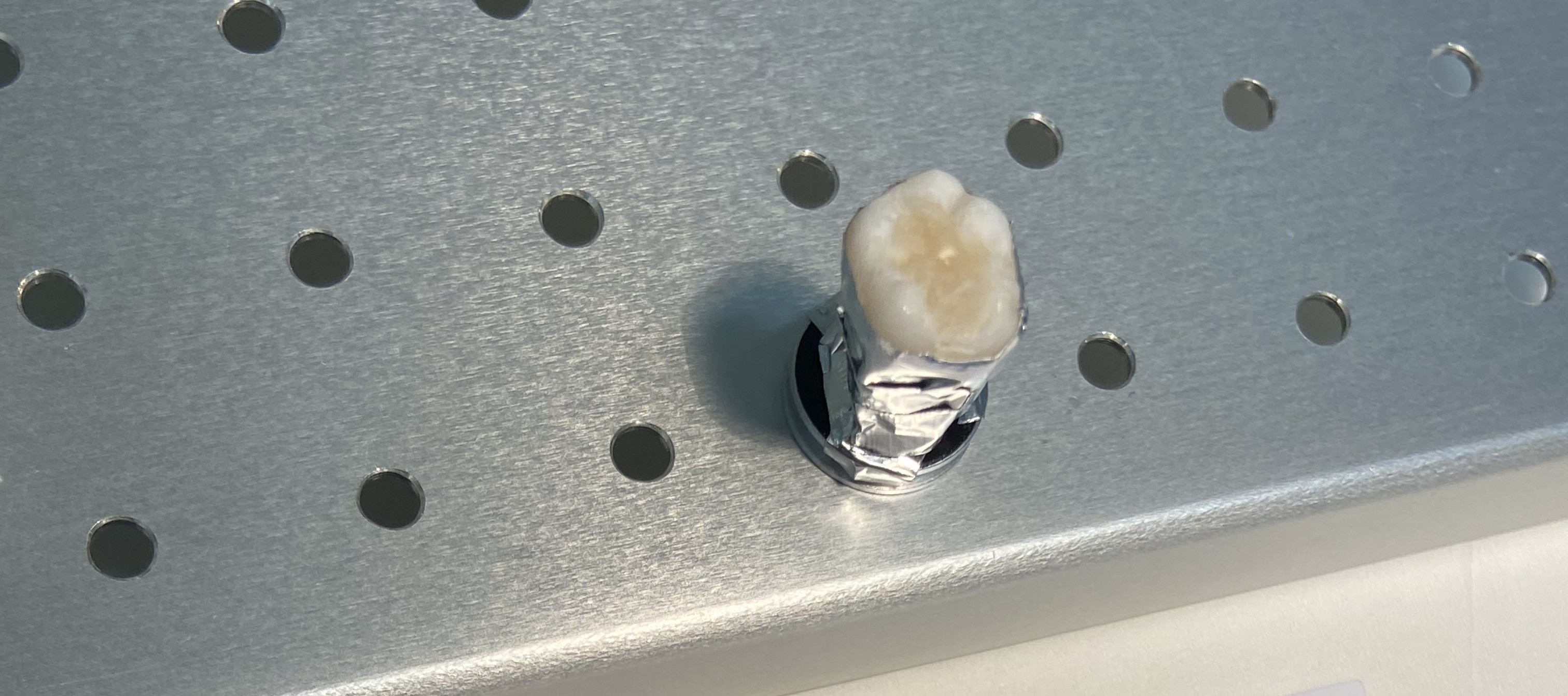

Supplement: Supplementary file 1 [file dentistry-14-00098-s001.zip › S5_SupplementaryFigures/FigureS6.tif]

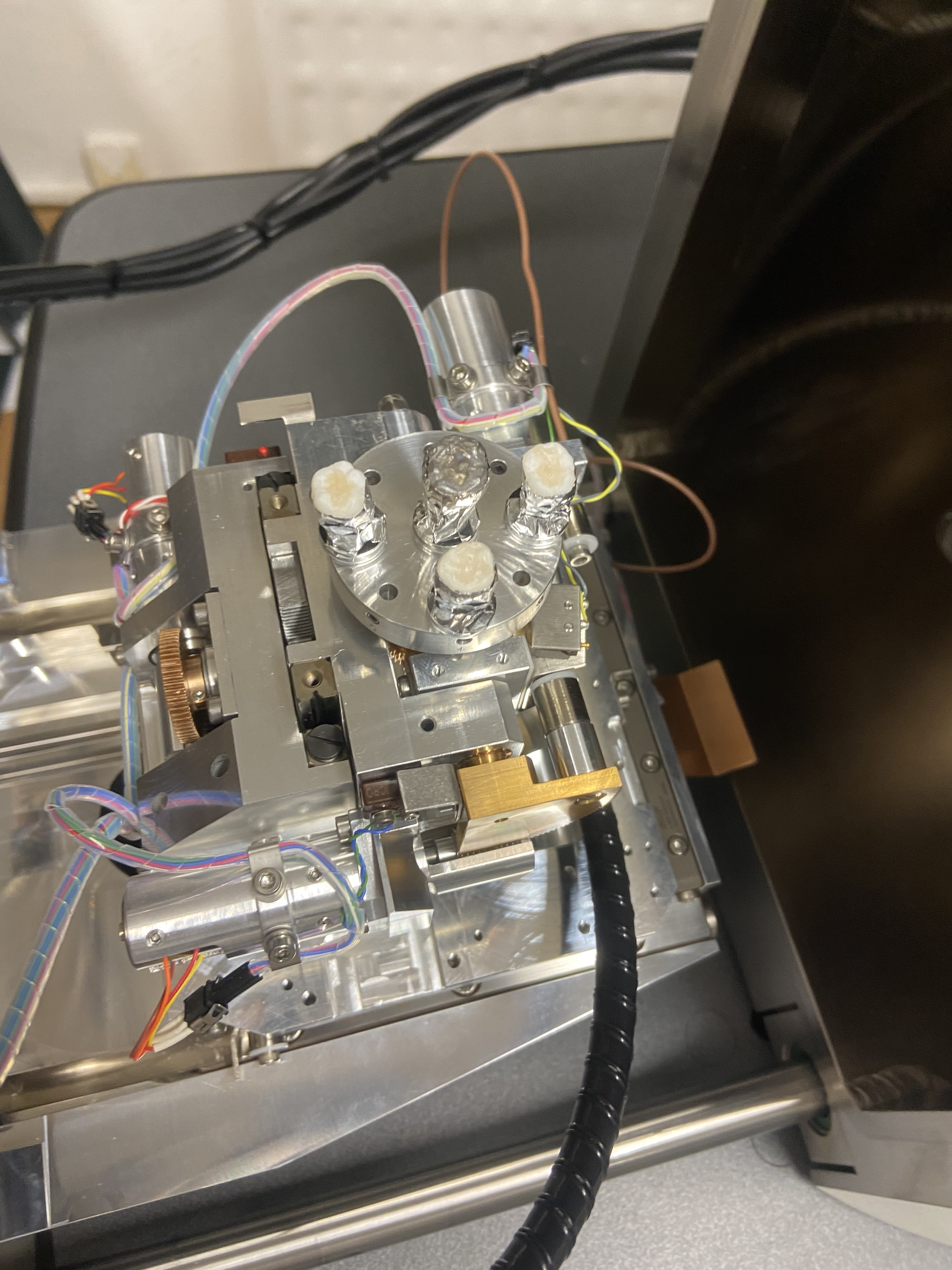

Supplement: Supplementary file 1 [file dentistry-14-00098-s001.zip › S5_SupplementaryFigures/FigureS7.tif]

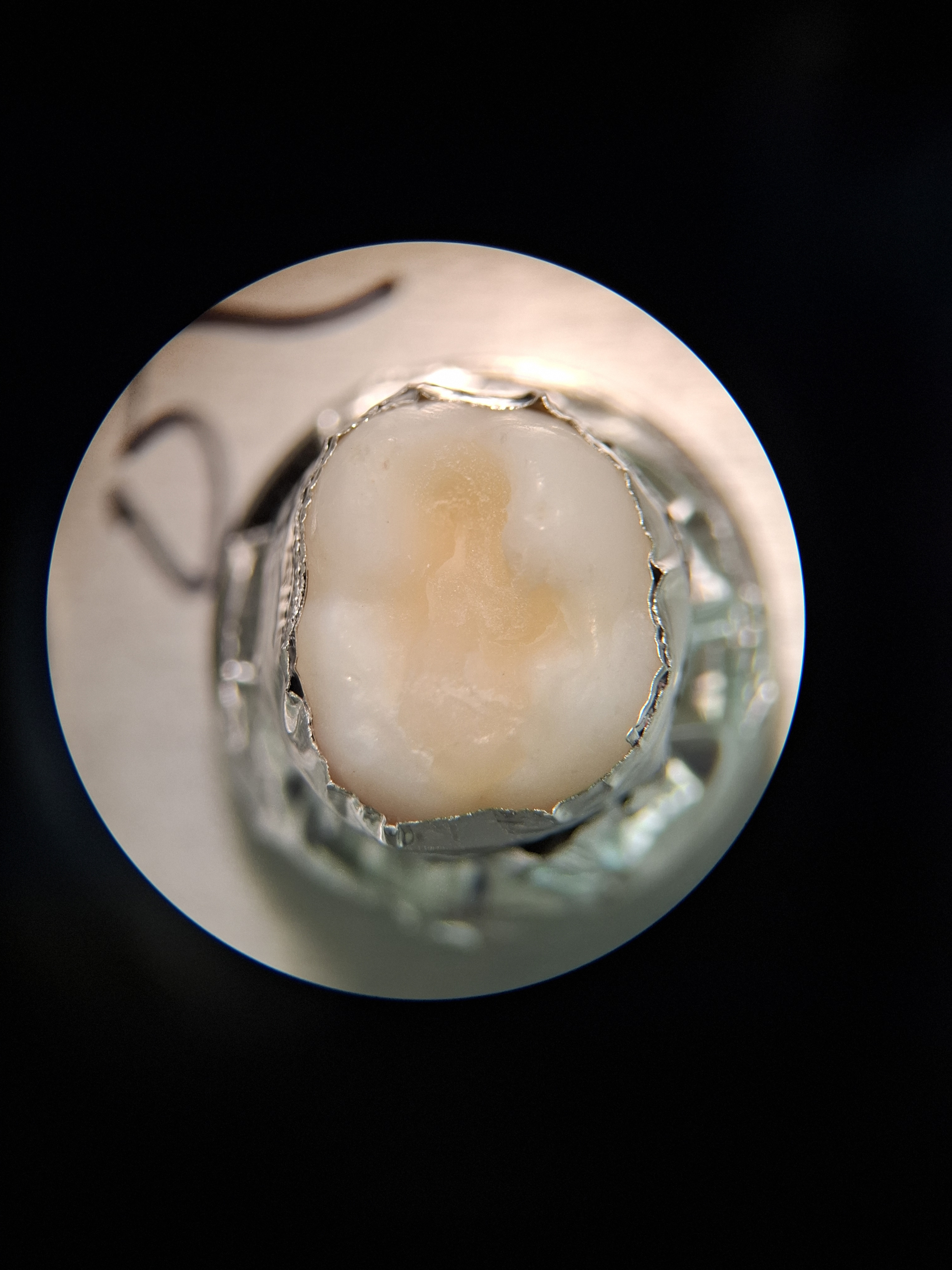

Supplement: Supplementary file 1 [file dentistry-14-00098-s001.zip › S5_SupplementaryFigures/FigureS8.tif]
